# The role of neuro-imaging in multiple system atrophy

**DOI:** 10.1007/s00702-025-02964-6

**Published:** 2025-07-12

**Authors:** Florian Krismer, Klaus Seppi, Werner Poewe

**Affiliations:** 1 Department of Neurology, Medical University Innsbruck, Anichstraße 35, 6020 Innsbruck, Austria; 2 Department of Neurology, Hospital Kufstein, Kufstein, Austria

**Keywords:** Multiple system atrophy, MSA, MRI, Neuro-imaging

## Abstract

Neuroimaging plays a crucial role in diagnosing multiple system atrophy and monitoring progressive neurodegeneration in this fatal disease. Advanced MRI techniques and post-processing methods have demonstrated significant volume loss and microstructural changes in brain regions well known to be affected by MSA pathology. These observations can be exploited to support the differential diagnosis of MSA distinguishing it from Parkinson's disease and progressive supranuclear palsy with high sensitivity and specificity. Longitudinal studies reveal aggressive neurodegeneration in MSA, with notable atrophy rates in the cerebellum, pons, and putamen. Radiotracer imaging using PET and SPECT has shown characteristic disease-related patterns, aiding in differential diagnosis and tracking disease progression. Future research should focus on early diagnosis, particularly in prodromal stages, and the development of reliable biomarkers for clinical trials. Combining different neuroimaging modalities and machine learning algorithms can enhance diagnostic precision and provide a comprehensive understanding of MSA pathology.

## Introduction

Multiple System Atrophy (MSA) is a rare neurodegenerative synucleinopathy characterized by neuronal loss and gliosis in multiple areas of the central nervous system including striatonigral, olivopontocerebellar and central autonomic structures (Poewe et al. 2022; Krismer et al. 2024a). Pathological diagnosis rests on the presence of oligodendroglial cytoplasmic inclusions containing misfolded and aggregated α-synuclein (Wiseman et al. 2025), while clinically established MSA requires the presence of autonomic dysfunction in combination with poorly levodopa-responsive parkinsonism and/or cerebellar ataxia (Wenning et al. 2022). Clinical diagnostic accuracy is suboptimal in early disease because of phenotypic overlaps with Parkinson disease or other types of degenerative parkinsonism or cerebellar disorders. A large clinicopathological series of 203 cases clinically diagnosed as MSA in life found alternative pathological diagnoses in 21% (Miki et al. 2019). MSA is a fatal disease with rapid progression and reduced life expectancy (Glasmacher et al. 2017; Poewe et al. 2022; Krismer et al. 2024a). However, rare cases of MSA patients with deviant clinical progression and prolonged survival have been reported and these cases may be difficult to diagnose clinically (Petrovic et al. 2012). Several neuroimaging modalities have been shown to support the differential diagnosis between MSA and ‘look alikes‘ including radiotracer imaging and a variety of MRI based approaches (Table 1).

**Table 1:** Imaging biomarkers used to study MSA

Target (technique and/or marker)	Findings in MSA	Diagnostic potential	Research setting	References
**Molecular imaging markers**				
Presynaptic nigrostriatal neuron integrity: different PET and SPECT tracers, which assess DDC activity (for example, ^18^F-DOPA PET), DAT availability (for example, ^123^I-ioflupane SPECT, ^99^mTc-TRODAT SPECT, ^18^F‐CFT PET) or VMAT‑2 activity (for example, ^11^C-DTBZ PET)	Decreased due to nigrostriatal dopaminergic denervation	Currently not fully established	Differential diagnosis versus non-MSA degenerative parkinsonism (based on distinct patterns of extra-striatal and striatal dopaminergic depletion as well as deep-learning-guided radiomic features and quantitative analysis including putamen, caudate and midbrain tracer binding); Early diagnosis of MSA-C (versus SAOA); Sensitive to disease progression; Potential marker for prediagnostic disease (for example, SAOA and RBD if combined with other yet to be defined biomarkers) Single day protocol for DaT-SPECT, ^18^F-FDG-PET and ^123^I-MIBG-szintigraphy in order to characterize neurodegenerative parkinsonism	Brooks et al. (2009), Iranzo et al. (2010), Meyer et al. (2017), Saeed et al. (2017), Poewe et al. (2017, 2022), Brumberg and Isaias (2018), Xu et al. (2018), Fanciulli et al. (2019), Pellecchia et al. (2020), Chougar et al. (2021), Peralta et al. (2022), Schröter et al. (2022), Zhao et al. (2022), Wan et al. (2023), Scheifele et al. (2025)
Striatal dopamine D2/D3 receptors: different PET and SPECT tracers (for example, ^123^I-IBZM SPECT, ^18^F-fallypride PET, ^11^C‐raclopride PET)	Binding decreased in putamen	Differential diagnosis versus PD (normal binding in PD), but not versus PSP; Overlapping individual values between PD and MSA Results may be confounded by several drugs (for example dopaminergic drugs or dopamine receptor	Differential diagnosis versus non-MSA degenerative parkinsonism (based on more sophisticated algorithms such as SVM analysis or influx analysis); Potential marker of disease progression; Potential marker for prediagnostic disease;	Brooks et al. (2009), Iranzo et al. (2010), Saeed et al. (2017), Poewe et al. (2017, 2022), Brumberg and Isaias (2018), Xu et al. (2018), Fanciulli et al. (2019), Pellecchia et al. (2020), Chougar et al. (2021), Peralta et al. (2022), Zhao et al. (2022), Wan et al. (2023), Scheifele et al. (2025)
Glucose metabolism (^18^F-FDG-PET)	hypometabolism in putamen, brainstem and cerebellum	early and differential diagnosis versus PD and with a lesser diagnostic accuracy versus PSP	MSARP is a diagnostic marker (versus non-MSA degenerative parkinsonism) and a potential marker for prediagnostic disease in MSA-P; Sensitive to disease progression; Potential surrogate marker for treatment effects; Single day protocol for DaT-SPECT, ^18^F-FDG-PET and ^123^I-MIBG-szintigraphy in order to characterize neurodegenerative parkinsonism	Brooks et al. (2009), Tang et al. (2010), Poston et al. (2012), Tripathi et al. (2016), Saeed et al. (2017), Poewe et al. (2017, 2022), Xu et al. (2018), Fanciulli et al. (2019), Pellecchia et al. (2020), Shen et al. (2020), Chougar et al. (2021), Schindlbeck et al. (2021), Peralta et al. (2022), Wan et al. (2023), Scheifele et al. (2025)
Microglial activation: different PET tracers (for example ^11^C(R)ePK11195-PET;^11^C-PBR28-PET)	Increased activation in prefrontal cortex, basal ganglia, pons, and substantia nigra	Currently not established; restricted to specialized research centers, thus far	Differential diagnosis versus PD (based on a sophisticated machine learning algorithm); Sensitive to disease progression; Potential surrogate marker for treatment effects	Brooks et al. (2009), Saeed et al. (2017), Poewe et al. (2017, 2022), Xu et al. (2018), Fanciulli et al. (2019), Kübler et al. (2019), Pellecchia et al. (2020), Chougar et al. (2021), Jucaite et al. (2022), Peralta et al. (2022), Wan et al. (2023(
Myocardial postganglionic sympathetic innervation: different tracers: for example ^123^I-MIBG szintigraphy/SPECT, ^11^C-metahydroxyephedrine PET, ^18^F-dopamine PET	Normal cardiac uptake, but decreased cardiac sympathetic innervation may occur	Differential diagnosis versus PD (decreased cardiac sympathetic innervation in PD; Overlapping individual values between PD and MSA; Results may be confounded by cardiac co-morbidity and drugs	Potential marker for prediagnostic disease if combined with other yet to be defined biomarkers (for example RBD, autonomic failure) Single day protocol for DaT-SPECT, ^18^F-FDG-PET and ^123^I-MIBG-szintigraphy facilitates biomarker assessments in order to characterize neurodegenerative parkinsonism	Brooks et al. (2009), Orimo et al. (2012), Saeed et al. (2017), Poewe et al. (2017, 2022), Brumberg and Isaias (2018), Goldstein and Cheshire (2018), Fanciulli et al. (2019), Pellecchia et al. (2020), Chougar et al. (2021), Eckhardt et al. (2022), Peralta et al. (2022), Wan et al. (2023)
α-synuclein PET imaging (e.g. ^18^F‐SPAL‐T‐06-PET; ^18^F-ACI-12589-PET)	Binding increased in putamen, pons, cerebellar white matter and middle cerebellar peduncles	Currently not established; restricted to specialized research centers, thus far	Differential diagnosis versus PD Potential marker for prediagnostic disease, characterization of subtypes of MSA, disease progression Potential marker for drug target engagement in vivo of novel α-synuclein targeting therapies	Matsuoka et al. (2022), Smith et al. (2023), Wan et al. (2023)
**Transcranial sonography**				
Nigral echogenicity (transcranial brain parenchyma sonography)	Normal nigral echogenicity (at least 10% have hyperechogenicity similar to patients with PD); lenticular nucleus hyperechogenicity	Early and differential diagnosis with suboptimal diagnostic accuracy versus PD (who have nigral hyperechogenicity in up to 90%); Not useful versus PSP	Potential marker for prediagnostic disease if combined with other yet to be defined biomarkers (for example presynaptic dopaminergic imaging, RBD, autonomic failure)	Walter et al. (2003), Behnke et al. (2005), Berg and Gaenslen (2010), Shafieesabet et al. (2017), Pellecchia et al. (2020), Poewe et al. (2022)
**Magnetic resonance Imaging**				
Brain structure (routine sequences of MRI)	Atrophy and signal alterations in putamen (hypointensity and lateral hyperintense rim), MCP (hyperintensity), pons (HCB) and cerebellum	Support differential diagnosis versus PD with high specificity and variable sensitivity (less sensitive in early disease stages); Suboptimal diagnostic accuracy versus PSP; Insufficient evidence for the differential diagnosis versus SAOA	Potential marker for prediagnostic disease; Potential surrogate marker for treatment effects	Brooks et al. (2009), Saeed et al. (2017), Poewe et al. (2017, 2022), Heim et al. (2018), Fanciulli et al. (2019), Pellecchia et al. (2020), Chougar et al. (2021, 2024), Peralta et al. (2022), Wan et al. (2023), Kawabata et al. (2024)
Brain structure (sophisticated volumetric analyses including for example gray matter volume analysis, cortical thickness measurements, cortical gyrification)	Specific atrophy pattern involving putamen, MCP, pons, cerebellum and cortical areas	Currently not established; restricted to specialized research centers, thus far	Differential diagnosis versus MSA mimics (based on machine learning approaches and automated volume segmentation on a single-patient level); Sensitive to disease progression (using automated methods for the longitudinal assessment of volumetric MRI); Potential surrogate marker for treatment effects; characterization of subtypes of MSA	Brooks et al. (2009), Scherfler et al. (2016), Saeed et al. (2017), Poewe et al. (2017, 2022), Heim et al. (2018), Fanciulli et al. (2019), Krismer et al. (2019, 2024b), Arribarat et al. (2020), Pellecchia et al. (2020), Zhang et al. (2020), Chougar et al. (2021, 2024), Peralta et al. (2022), Chen et al. (2023b), Rau et al. (2023), Schröter et al. (2024)
Iron accumulation (different metrics of specific iron-sensitive sequences: for example R2* with GRE sequences; phase shift values, signal intensities or iron percentage with SWI; susceptibility values with QSM)	Increased putaminal iron content (mainly in the posterior part of the putamen)	Differential diagnosis versus PD, suboptimal versus PSP; Insufficient evidence for the differential diagnosis versus SAOA	Quantification of topographical differences of iron accumulation might increase diagnostic accuracy versus MSA mimickers	Brooks et al. (2009), Saeed et al. (2017), Poewe et al. (2017, 2022), Heim et al. (2018), Fanciulli et al. (2019), Lee and Lee 2019), Pellecchia et al. (2020), Chougar et al. (2021, 2023, 2024), Lancione et al. (2022), Pang et al. (2022), Peralta et al. (2022), Wan et al. (2023), Yan et al. (2024)
Neuromelanin content (NM-sensitive MR sequences)	Reduced size and signal intensity in SN and LC	Insufficient diagnostic accuracy versus non-MSA degenerative parkinsonism; Insufficient evidence for the differential diagnosis versus SAOA	Investigational as diagnostic marker because of possible topographical differences of NM-content versus MSA mimickers	Fanciulli et al. (2019), Arribarat et al. (2020), Pellecchia et al. (2020), Simões et al. (2020), Chougar et al. (2021, 2022, 2024), Peralta et al. (2022), Poewe et al. (2022), Nobileau et al. (2023), Wan et al. (2023), Pasquini et al. (2025)
Brain microstructure (different diffusion metrics assessed with diffusion imaging including MD, FA, AD, FW, FA_T_)	Abnormal nigral (only FW), putaminal and MCP diffusion metrics	Differential diagnosis versus non-MSA degenerative parkinsonism, diagnostic accuracy better versus PD than versus PSP; Insufficient evidence for the differential diagnosis versus SAOA	Early diagnostic marker; Improvement of differential diagnosis versus MSA mimickers using AID-P; Sensitive to disease progression	Brooks et al. (2009), Saeed et al. (2017), Poewe et al. (2017, 2022), Heim et al. (2018), Archer et al. (2019), Fanciulli et al. (2019), Pellecchia et al. (2020), Beliveau et al. (2021), Chougar et al. (2021, 2023, 2024), Krismer et al. (2021), Peralta et al. (2022), Vaillancourt et al. (2025)
Complementary brain tissue changes (multimodal MRI)	Abnormal putaminal and MCP diffusion metrics, abnormal putaminal iron content, abnormal brainstem volumetric/planimetric measures, abnormal putamen volumetric measures; abnormal functional activity of the dorsolateral putamen	Currently not established; restricted to specialized research centers, thus far	Diagnostic (early diagnosis) marker versus MSA mimickers; Harmonization of MRI protocols and analysis platforms	Saeed et al. (2017), Péran et al. (2018), Fanciulli et al. (2019), Nemmi et al. (2019), Arribarat et al. (2020), Pellecchia et al. (2020), Chougar et al. (2021, 2024), Pang et al. (2022), Peralta et al. (2022), Poewe et al. (2022), Chen et al. (2023b), Wan et al. (2023)
Structural connectivity with tractography (for example diffusion metrics within the tracts; number of tracks; connection probability between regions)	Altered structural connectivity in both basal ganglia and cerebellar connectivity	Currently not established; restricted to specialized research centers, thus far	Diagnostic marker; Characterization of subtypes of MSA	Fukui et al. (2016), Abos et al. (2019), Shah et al. (2019), Faber et al. (2020), Pellecchia et al. (2020), Beliveau et al. (2021), Poewe et al. (2022), Wan et al. (2023), Chougar et al. (2024)
functional connectivity with rs-fMRI (for example correlation coefficient; integration what quantifies how signals covary between regions belonging to a particular network; small-world network indices), ASL (for example rCBF or CMRO2) or voxel-wise fractional amplitude of low-frequency fluctuation followed by seed-based functional connectivity (spontaneous brain activity)	Altered coupling in different basal ganglia, cerebellar and central autonomic network brain networks; Functional connectivity disruptions mainly in the cortico-thalamo-cerebellar circuits within the cerebellum-basal ganglia-cortical network in MSA-P Spontaneous brain activity within basal ganglia, cerebellum, and cortices in early-stage MSA-P using	Currently not established; restricted to specialized research centers, thus far	Diagnostic marker; Characterization of subtypes of MSA	Pellecchia et al. (2020), Poewe et al. (2022), Chen et al. (, 2023a, b), Lyu et al. (2023), Wang et al. (2024), Li et al. (2025)

*AID-P* Automated Imaging Differentiation in Parkinsonism using machine learning approaches and FW/FAT of regions/tracts within the basal ganglia cerebellum and cortex, *AD* axial diffusivity, *ASL* Arterial Spin Labeling, *rCBF* regional cerebral blood flow, *CMRO2* cerebral metabolic rate of oxygen consumption, *CFT* 2b-carbomethoxy-3b-(4-fluorophenyl)tropane, *DAT* dopamine transporter, *DaT-SPECT* ^123^I-ioflupane SPECT, *DDC* dopa decarboxylase, *DTBZ* dihydrotetrabenazine, *FW* free water, *FA*_*T*_ FW-corrected FA, *HCB* hot cross bun sign, *IBZM* iodobenzamide, *MCP* middle cerebellar peduncle, *MD* mean diffusivity, *MIBG* metaiodobenzylguanidine, *MSA* multiple system atrophy, *MSARP* MSA related spatial covariance pattern based on global functional level spatial covariance analysis of metabolic alterations, *LC* locus coeruleus, *NM* neuromelanin, *PD* Parkinson disease, *PSP* progressive supranuclear palsy, *QSM* quantitative susceptibility mapping, *RD* radial diffusivity, *RBD* REM sleep behavior disorder, *rs-fMRI* resting state functional MRI, *SAOA* sporadic late-onset ataxias, *SN* substantia nigra, *SVM* support vector machine, *TCS* transcranial brain parenchyma sonography, *TRODAT* 2[[2-[[[3-(4-chlorophenyl)-8-methyl-8-azabicyclo[321]-oct-2-yl]-methyl](2-mercaptoethyl) amino]ethyl]amino]ethane-thiolato(3-)-N2N20S2S2]oxo-[1R-(exo-exo), *VMAT-2* vesicular monoamine transporter 2

Structural routine MRI may enhance clinical diagnostic precision and pathological MRI features have been added as a requirement for a diagnosis of ‘clinically established MSA’ in the recent MDS diagnostic criteria for MSA (Wenning et al. 2022). Neuroimaging using advanced MR techniques including automated volumetric analysis, diffusion tensor and multimodal imaging have the potential to significantly further enhance early differential diagnosis (Chougar et al. 2024).

Here we review the role of neuroimaging modalities in early and differential diagnosis as well as their performance as progression markers that can be employed in trials of disease-modifying interventions in MSA. The interest of our group in this area of clinical MSA research was sparked off by Gregor Wenning more than 20 years ago, when he first suggested to explore diffusion MRI as a diagnostic tool in atypical parkinsonian disorders (Seppi et al. 2003).

## Role of imaging in the diagnosis of MSA

**Fig. 1:**
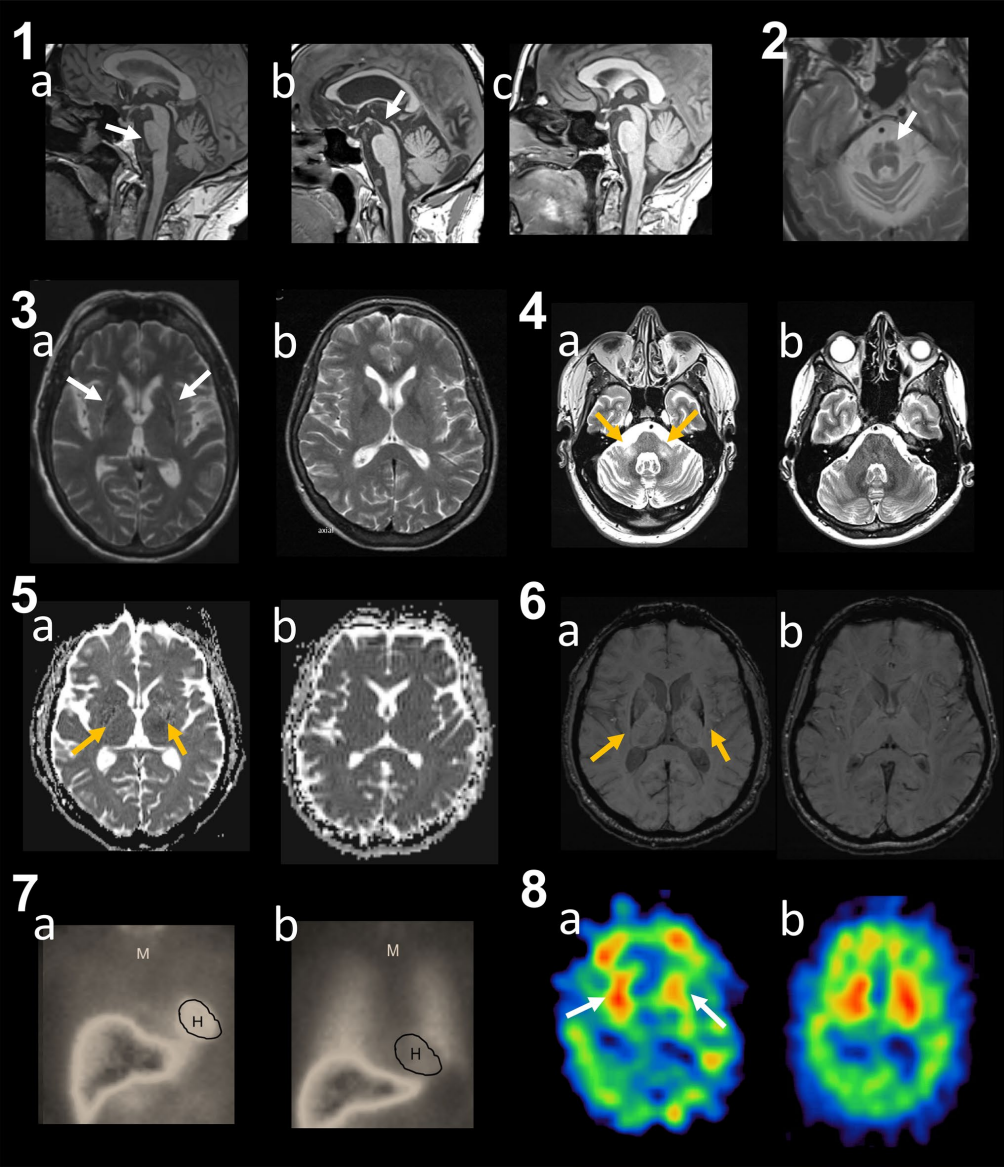
Imaging methods used study MSA. Panel 1: Midsagittal T1-weighted images showing Infratentorial atrophy (pons and cerebellum; dilated forth ventricle) (arrow e pons) in a patient with MSA (**a**) and the hummingbird sign (arrow) (atrophy of the rostral midbrain tegmentum) in a patient with PSP (**b**), while there is no relevant brainstem atrophy in a patient with PD (C). Panel 2: “Hot cross bun” sign (arrow) in a patient with MSA on T2-weighted images. Panel 3: Putaminal changes (atrophy, hyperintense rim, putaminal hypointensity in comparison with the globus pallidus) (arrows) at both sides in a patient with MSA (**a**) on T2-weighted images compared to a patient with PD (**b**) having no basal ganglia abnormalities. Panel 4: atrophy of MCP with the MCPsign (hyperintensity in the MCP) (arrows) on T2-weighted images in a patient with MSA (**a**) compared to a PD patient (**b**). Panel 5: Note the diffuse hyperintensity (corresponding to increased diffusivity values) in the posterior part of both putamina (arrows) in a patient with MSA (**a**) compared to a PD patient (**b**) on DWI. These changes in the MSA patient were observed only 6 months after onset of levodopa responsive parkinsonism with an anticipation of 18 months in relation to the clinical diagnosis of possible MSA-P and of 24 months for the diagnosis of probable MSA. Panel 6: Putaminal atrophy and putaminal hypointensity (arrows) on SWI in a patient with MSA (**a**) compared to a PD patient (**b**). As in this MSA patient, putaminal hypointensity start typically in the dorsolateral part of the putamen. Panel 7: Planar cardiac delayed 123I-MIBG imaging in a patient with MSA (**a**) compared with a patient with early PD (**b**). There is markedly reduced MIBG uptake in the heart (H) in the patient with PD compared to the mediastinum (**m**). Panel 8: Post-synaptic dopaminergic imaging with 123I-IBZM SPECT shows normal striatal tracer uptake in a PD patient (**b**), while in the MSA (**a**) patient there is reduced asymmetric striatal (arrows) tracer uptake with more marked reduction in the left. *DWI* diffusion-weighted imaging, *IBZM* iodobenzamide, *MIBG* metaiodobenzylguanidine, *MCP* middle cerebellar peduncle, *MSA* multiple system atrophy, *PD* Parkinson’s disease, *SWI* susceptibility-weighted imaging. This figure is replicated with permission from Fanciulli et al. (2019), Elsevier

Different neuroimaging modalities have been studied as potential surrogate markers of underlying neurodegeneration in MSA reflecting cell loss in affected brain regions, altered microstructural integrity as well as reactive microglial proliferation and astroglial activation. Brain volume can be quantified using MRI through several, rater-dependent as well as automated and semi-automated methods. These methods typically involve segmentation of the brain into different tissue types, such as gray matter (GM), white matter (WM), and cerebrospinal fluid (CSF), followed by volumetric analysis. Conventional MRI in MSA may show a variety of features with greater than 80% specificity but limited sensitivity for MSA including putaminal atrophy, atrophy of the pons, middle cerebellar peduncle (MCP), medulla oblongata and cerebellum as well as T2 signal intensity changes with putaminal hypointensity, MCP hyperintensity and the “hot cross bun sign” in the pons (see Fig. 1) (Heim et al. 2018; Pellecchia et al. 2020). Diffusion-weighted MRI sequences may show increased diffusivity in the posterior putamen and MCP and iron-sensitive MRI sequences like T2* or quantitative susceptibility mapping (QSM) can detect abnormal iron accumulation in the posterior region of the putamen, the globus pallidus as well as S. nigra and dentate nucleus and when present these findings have high specificity for MSA (Wang et al. 2012; Bajaj et al. 2017; Nobileau et al. 2023).

Automated brain segmentation from volumetric T1-weighted MRI have consistently shown high sensitivity and specificity for the differentiation of MSA from its most common mimics PD or PSP (Scherfler et al. 2016; Krismer et al. 2019). Using individual voxel-based morphometry adjusting for covariates (iVAC), Ebina et al. have reported 95% sensitivity and 96% specificity for MSA-P versus PD based on volume loss in the pons/cerebellum and putamen (Ebina et al. 2021). Machine learning algorithms enable observer independent analyses of MSA specific patterns of volume loss involving the putamen, cerebellum and MCP resulting yielding high diagnostic accuracy for MSA versus PD or PSP (Scherfler et al. 2016; Krismer et al. 2019; Rau et al. 2023; Chougar et al. 2024).

Recent studies have highlighted the utility of diffusion imaging in differentiating MSA from related parkinsonian diseases, particularly Parkinson's disease (Pasquini et al. 2022). Widespread white matter abnormalities in subcortical structures like the putamen as well as in the middle cerebellar peduncle were commonly reported (Pasquini et al. 2022). In fact, a meta-analysis of manual region-of-interest-based measurements of putaminal diffusivity demonstrated an overall sensitivity of 90% and an overall specificity of 93% for separating MSA from Parkinson’s disease based on a limited number of patients with established disease (Bajaj et al. 2017). Fully automated localization and analysis of mean diffusivity and fractional anisotropy in the MCP and putamen yielded an overall diagnostic accuracy of 91.4% for distinguishing MSA from Parkinson’s disease (Krismer et al. 2021). This observer-independent approach addresses the variability in and training needs required for manual analyses and, thus, likely enhances reproducibility. Similarly, tractography and diffusion-tensor imaging (DTI) have been shown to enhance diagnostic accuracy; one such study has recently demonstrated that DTI metrics in the MCP and putamen can accurately distinguish MSA from Parkinson’s disease, achieving 91.7% overall accuracy in differentiating the two conditions (Beliveau et al. 2021). The inclusion of putaminal volume further improved diagnostic accuracy to 95.8%, underscoring the potential of multimodal MRI to enhance diagnostic precision (Beliveau et al. 2021). Advanced diffusion imaging techniques, such as fixel-based analysis and free-water imaging, have also been employed in an attempt to discriminate MSA from related diseases (Pasquini et al. 2022). Free-water imaging, a post-processing technique applied to diffusion MR, in particular, has shown promise in detecting microstructural changes by separating the free-water component from tissue water, thus providing a more precise measure of tissue integrity. Planetta et al. (2016) found that free-water values were increased in several brain regions affected by MSA pathology, such as the putamen and middle cerebellar peduncle, hinting at disease-specific patterns of free water abnormalities in MSA (Planetta et al. 2016). A subsequent multi-centre study aimed to develop and validate an automated imaging approach, called „Automated Imaging Differentiation in Parkinsonism “ (AIDP), to differentiate parkinsonian diseases based on free water imaging (Archer et al. 2019). The study, conducted at 17 academic centres, involved 1002 patients and used machine learning models to analyze free water metrics in 60 brain regions. The results showed that diffusion imaging could accurately distinguish Parkinson's disease from atypical parkinsonism as well as MSA from progressive supranuclear palsy (Archer et al. 2019). A prospective multicentre cohort study has recently confirmed the high accuracy of the AIDP in distinguishing MSA from related parkinsonian diseases (Vaillancourt et al. 2025). The area under the receiver operating characteristic curve for differentiating MSA from Parkinson’s disease was 0.98, indicating excellent discriminative performance (Vaillancourt et al. 2025). Additionally, the AIDP was robust in differentiating progressive supranuclear palsy from MSA, with an area under the receiver operating characteristic curve of 0.98 (Vaillancourt et al. 2025). In summary, DTI and its advanced methods, including tractography, fixel-based analysis, and free-water imaging, offer significant diagnostic potential in MSA.

Transcranial sonography imaging as a cheap structural imaging method may also be helpful in differentiating MSA from PD, as hyperechogenicity in the midbrain area is usually found in up to 90% of PD cases but is absent in most patients with MSA (Walter et al. 2003; Behnke et al. 2005; Berg and Gaenslen 2010; Poewe et al. 2022). In fact, a meta-analysis revealed a sensitivity of 83% (95% CI 65–93%) and a specificity of 70% (95% CI 41–89%) of midbrain hyperechogenicity in distinguishing PD from MSA (Shafieesabet et al. 2017). Notably, up to 15% of patients cannot be assessed with transcranial sonography (Pellecchia et al. 2020) and there is a lack of studies addressing its use to distinguish MSA from other mimics.

Dopaminergic radiotracer imaging using dopamine transporter (DAT)-single photon emission computed tomography (SPECT) or cardiac 123I-MIBG-SPECT typically show loss striatal dopaminergic tracer uptake and preserved cardiosympathetic innervation in MSA, however, their usefuleness in differentiating MSA from PD or other types of degenerative parkinsonism is limited. Reductions in striatal DAT binding are not specific to MSA and cannot distinguish between different types of degenerative parkinsonism and may also be abnormal in some degenerative cerebellar ataxias like SCA 2 (Scherfler et al. 2007). While a finding of preserved cardiac sympathetic innervation using 123I-MIBG-SPECT or 18F-dopamine PET imaging as compared to a reduced tracer uptake indicating myocardial postganglionic sympathetic dysfunction in PD or DLB can support a clinical diagnosis of MSA (Orimo et al. 2012), normal tracer uptake in 123I-MIBG-SPECT has been reported in up to 60% of subjects with early PD and up to 44% of MSA subjects have been found to show normal cardiac-to-mediastinum binding ratios (Köllensperger et al. 2007; Catalan et al. 2021; Eckhardt et al. 2022). Adding to these limitations in specificity sympathetic cardiac imaging is vulnerable to confounding factors like comorbid cardiomyopathy, reductions in cardiac sympathetic innervation due to autonomic neuropathies or effects of medications that can interfere with tracer binding (Rissardo and Caprara 2023).

Metabolic radiotracer imaging using 18F-FDG-PET has been shown to reveal characteristic disease related patterns in degenerative parkinsonism like PD, PSP or MSA with hypometabolism in the putamen, MCP, pons and cerebellum characterizing MSA (Brooks et al. 2009; Tang et al. 2010; Poston et al. 2012; Tripathi et al. 2016; Meyer et al. 2017; Saeed et al. 2017; Poewe et al. 2017, 2022; Xu et al. 2018; Fanciulli et al. 2019; Pellecchia et al. 2020; Shen et al. 2020; Chougar et al. 2021; Schindlbeck et al. 2021; Peralta et al. 2022; Schröter et al. 2022; Wan et al. 2023; Scheifele et al. 2025). Automated image-based analysis has been used to enable individual patient diagnostic classification in a cohort of 167 subjects with PD, MSA or PSP and sensitivity and specificity for a clinical MSA diagnosis were 85% and 96% respectively (Tang et al. 2010). A comparison between 18F-FDG-PET and 123I-MIBG-SPECT could not identify statistically significant differences in diagnostic accuracy (Brumberg et al. 2020). More recently, glial imaging using the TSPO ligand 11C-PBR28 and a machine learning algorithm revealed high diagnostic accuracy in discriminating MSA from PD based on a pattern of elevated regional tracer binding in MSA involving the lentiform nucleus and cerebellar white matter (Jucaite et al. 2022).

Despite the growing recognition of neuroimaging markers as valuable tools for facilitating early diagnosis in subjects in prodromal or premanifest stages of PD (Höglinger et al. 2024; Scheifele et al. 2025), there remains a paucity in data on the performance of neuroimaging in the detection of MSA in its prodromal stages. Of note, recently implemented diagnostic criteria for possible prodromal MSA require either polysomnography-proven REM-sleep behavior disorder or isolated autonomic failure as an entry criterion for prodromal MSA (Wenning et al. 2022). A longitudinal volumetric MRI study has suggested that pontine atrophy may precede clinical manifestation in MSA-C (Kawabata et al. 2025), but this was based on retrospective modelling of the observed trajectories in clinically diagnosed subjects. Efforts to develop alpha-synuclein PET tracers have yielded promising results in MSA patients with robust tracer uptake in the striatum and infratentorial regions (see ‘future perspectives’ section below).

## Use of neuroimaging to track disease progression

Voxel-based morphometry (VBM) is applied to groups of scans to highlight atrophy patterns between a group of patients and within individual patients over time. By contrast, methods using registration of serial imaging are applied to individual scan pairs with accurate registration of follow-up and baseline scans, thus determining rates of atrophy rather than patterns.

In one study, VBM was applied to a series of MR images in 14 patients with MSA with baseline and follow-up scans being one year apart. This study demonstrated marked progression of brain atrophy in the putamen, midbrain, thalamus, cerebellum and several cortical regions (Brenneis et al. 2007). To exclude an age effect and confirm the more aggressive neurodegeneration in MSA compared with Parkinson’s disease, the same VBM approach was employed in an age-matched PD cohort, which showed no progression of brain atrophy (Brenneis et al. 2007). Additionally, shorter disease duration correlated with progression of atrophy in the striatum, whereas longer disease duration correlated with increasing atrophy in the cortical areas and cerebellar hemispheres, suggesting that degeneration of the basal ganglia occurs early in the disease process (Brenneis et al. 2007). Another study investigated the rates and regions of brain atrophy in MSA (n = 11), progressive supranuclear palsy (n = 24), Parkinson's disease (n = 12) and healthy controls (n = 18). MSA patients had the highest rate of pontine atrophy at 4.5% per year, more than 20 times higher than healthy controls and three times higher than PSP patients. Cerebellar atrophy rates in MSA were also significant at 3.2% per year, distinguishing MSA from PSP and healthy controls (Paviour et al. 2006). More recently, a longitudinal, multi-centre study using observer-independent brain morphometry has demonstrated significant brain volume loss in MSA patients over a 12-month period in the cerebellar cortex, cerebellar white matter, pons and putamen; all of which are regions known to be affected by MSA pathology (Krismer et al. 2024b). The annual atrophy rates observed in this study ranged from 4.2 to 8.2%, which is significantly higher than the rates observed in Parkinson’s disease patients and healthy controls. These findings underscore the aggressive nature of neurodegeneration in MSA and support the potential use of MRI morphometry as a surrogate marker of disease progression in this fatal disease (Krismer et al. 2024b). A further long-term study with bi-annual MRI scans over a period of four years investigated the progression of atrophy in patients diagnosed with cerebellar-type MSA. The study revealed non-linear alterations in pontine volumes, characterized by an initial phase of progressive atrophy in early disease stages, subsequently transitioning to a plateau phase in late disease (Kawabata et al. 2025). Statistical modelling indicated the onset of pontine atrophy in pre-motor disease (Kawabata et al. 2025).

A further study investigated multimodal imaging progression with assessment of subcortical atrophy on T1-weighted images and iron deposition on R2* images in a cohort of 17 MSA patients and 15 patients with Parkinson's disease (Lee et al. 2015). This study found that both volume and R2* values exhibited a higher rate of progression in Parkinson-type MSA patients compared to MSA-C patients and Parkinson's disease. Specifically, the putamen and caudate nucleus exhibited more pronounced atrophy in MSA-P, and the R2* values in the putamen were significantly higher in MSA-P patients (Lee et al. 2015). Diffusion imaging has also been instrumental in detecting and quantifying early microstructural changes in MSA. Comparing progression of longitudinal diffusion changes in the basal ganglia of MSA-P versus PD patients one study found a marked increase of putaminal diffusivity over time in the MSA-P cohort which correlated with motor progression as assessed with the UPDRS-III (Seppi et al. 2006). Pellecchia et al. conducted a longitudinal diffusion imaging study that revealed significant increases in diffusivity in the putamen, pons, cerebellar white matter, thalamus, and frontal white matter over one year in a cohort of MSA-P and MSA-C patients (Pellecchia et al. 2011). Supporting this observation, a group of researchers analyzed two serial multimodal MRIs taken approximately 23 months apart from 12 MSA patients and 18 age- and sex-matched controls as well as PSP patients (Reginold et al. 2014). The study’s key findings include greater volume loss in the pons in MSA patients over time compared to controls and marked changes in ADC values suggesting that diffusivity measures of tissue microstructure may serve as a better biomarker than volumetric measures of tissue macrostructure in tracking disease progression (Reginold et al. 2014). Along those lines, Vemuri and colleagues studied morphometric changes and diffusion measures in 29 MSA patients with longitudinal changes in brainstem and pons volumes found to be significant in both MSA-C and MSA-P, indicating the involvement of these regions in disease progression (Vemuri et al. 2022). Further, diffusion measures in cerebellar white matter were also found to be sensitive indicators of neurodegeneration and diffusion metrics demonstrated superiority over volume measures (Vemuri et al. 2022).

Furthermore, the ratio between the signal intensity of T1-weighted and T2-weighted structural MRI images (T1w/T2w) has been proposed as a surrogate measure of myelin and iron content as well as axon and dendrite density (Glasser and Essen 2011; Arshad et al. 2017; Ponticorvo et al. 2021). A recent longitudinal study has demonstrated a significant increase in the T1w/T2w ratio within the cerebellar and left putaminal gray matter, as well as a decrease in the substantia nigra of patients diagnosed with MSA (Ponticorvo et al. 2022).

Overall, these findings further support the notion that quantitative MRI analysis has the capacity to function as both a diagnostic biomarker and a surrogate marker for disease progression in early MSA. Putative sample size estimates based on established progression rates of clinical and neuroimaging markers are presented in Table 2.

**Table 2:** Sample size estimates based on studies reporting progression rates of clinical and neuroimaging markers

Reference	Study type	Category	Measure	Mean annual change	SD	N needed
Low et al. (2015)	Observational	Clinical Score	UMSARS Total	7.58	8.39	288
Wenning et al. (2013)	Observational	Clinical Score	UMSARS Total	14.60	11.80	154
Levin et al. (2019)	RCT	Clinical Score	UMSARS Total	10.35	11.47	288
Low et al. (2014)	RCT	Clinical Score	UMSARS Total	10.80	10,70	231
Poewe et al. (2015)	RCT	Clinical Score	UMSARS Total	7.80	10.44	419
Krismer et al. (2024b)	Mixed	Imaging	Atrophy, Pons	4.67	4.88	256
Krismer et al. (2024b)	Mixed	Imaging	Atrophy, Putamen	4.25	4.87	308
Paviour et al. (2007)	Observational	Imaging	Atrophy, Cerebellum	3.20	1.90	84
Paviour et al. (2007)	Observational	Imaging	Atrophy, Pons	4.50	3.20	120
Vemuri et al. (2022)	Observational	Imaging	Atrophy, Pons	Not reported assumed effect size 0.4886, based on reported sample size estimate		90
Vemuri et al. (2022	Observational	Imaging	Atrophy, Putamen	Not reported assumed effect size 0.3171, based on reported sample size estimate		210
Nocker et al. (2012)	Observational	Imaging	Dopamine Transporter, Caudate	0.26	0.19	126
Nocker et al. (2012)	Observational	Imaging	Dopamine Transporter, Putamen	0.17	0.18	263
Vemuri et al. (2022)	Observational	Imaging	Mean diffusivity, Cerebellum WM	Not reported assumed effect size 0.4002, based on reported sample size estimate		133
Pellecchia et al. (2011)	Observational	Imaging	Mean Diffusivity, Putamen	0.09	0.05	83
Levin et al. (2019)	RCT	Imaging	Atrophy, Cerebellum	3.40	3.70	278
Palma et al. (2022)	RCT	Imaging	Atrophy, Cerebellum	1.17	0.73	92
Palma et al. (2022)	RCT	Imaging	Atrophy, Left Putamen	8.26	2.91	30
Levin et al. (2019)	RCT	Imaging	Atrophy, Pons	5.80	4.30	130
Palma et al. (2022)	RCT	Imaging	Atrophy, Pons	4.69	2.26	56
Levin et al. (2019)	RCT	Imaging	Atrophy, Putamen	0,07	0,05	121
Poewe et al. (2015)	RCT	Imaging	Mean Diffusivity, Putamen	23,06	12,61	71

The required sample size for detecting a statistically significant differences between two independent groups was estimated using the pwr.t.test function in R (R version 4.5.0, package ‘pwr’). The analysis was based on a two-sided, two-sample t-test assuming a 30% effect of an intervention, a desired statistical power of 90% and a significance level of 0.05. In case of RCTs, progression rates of the control group were considered

*RCT* randomized controlled trial, *SD* standard deviation, *UMSARS* unified MSA Rating Scale

Longitudinal radiotracer imaging studies have been conducted in MSA patients as well, targeting different neurochemical systems. The most commonly used tracers in positron emission tomography (PET) and single-photon emission computed tomography (SPECT) imaging target presynaptic dopamine integrity, glucose metabolism and microglial activation. One study characterized the progression of dopamine transporter (DAT) decline using 123I-Beta-CIT in the striatum and extrastriatal regions, including the midbrain and pons of patients with the Parkinsonian variant of MSA (Nocker et al. 2012). In this study, a relative decline of tracer uptake at follow-up compared with the baseline scan was evident in the caudate and anterior putamen of MSA-P versus Parkinson's disease (PD) (Nocker et al. 2012). While there were no large-scale, longitudinal 18F-FDG-PET studies in MSA, evidence from cross-sectional studies suggest that the pattern of glucose metabolism correlates with disease severity and disease duration in MSA (Poston et al. 2012). Microglial activation has been examined using 11C-PK11195-PET and more recently 11C-PBR28-PET and (Dong et al. 2025). Intriguingly, PK11195 PET has been used as an exploratory outcome measure in a subgroup of participants in the 'Minocycline for MSA' trial (Dodel et al. 2010) and a phase II study of the myeloperoxidase inhibitor Verdiperstat (clinicaltrials.gov identifier NCT02388295). Although the progression of microglial activation in the placebo arm of the Minocycline trial revealed a heterogeneous pattern of changes in different brain regions, there was an increase in the sum of 11C-PK11195 binding in the placebo arm over the 12-month study period (Dodel et al. 2010). 11C-PBR28 is a second-generation TSPO PET tracer that offers improved sensitivity and specificity for detecting microglial activation and 11C-PBR28 has demonstrated increased microglial activation in MSA patients, correlating with disease severity and progression (Jucaite et al. 2022).

## Conclusions and future perspectives

In summary, neuroimaging play a pivotal role in diagnosing and monitoring disease progression in MSA (see Table 1). In particular, recent reports demonstrated that automated brain segmentation and machine learning algorithms have a high sensitivity and specificity in distinguishing MSA from Parkinson's disease and progressive supranuclear palsy by identifying patterns of volume loss in specific brain regions such as the putamen, cerebellum, and middle cerebellar peduncle (MCP) and microstructural changes on diffusion imaging (Ofori et al. 2017; Péran et al. 2018; Archer et al. 2019; Nemmi et al. 2019; Krismer et al. 2019, 2021; Beliveau et al. 2021; Chougar et al. 2024; Vaillancourt et al. 2025). Longitudinal studies employing various neuroimaging modalities, including diffusion imaging, voxel-based morphometry (VBM), and radiotracer imaging, have shown significant brain volume loss and microstructural changes in MSA patients over time. These changes are more pronounced in MSA compared to PD, highlighting the aggressive nature of MSA. Quantitative MRI analysis has emerged as a potential diagnostic biomarker and surrogate marker for disease progression in MSA (Paviour et al. 2006; Reginold et al. 2014; Vemuri et al. 2022; Krismer et al. 2024b), with diffusion measures appearing to be superior to volume measures in tracking neurodegeneration (Vemuri et al. 2022). Additionally, metabolic radiotracer imaging using 18F FDG-PET and glial imaging with TSPO ligands have revealed characteristic disease-related patterns in MSA, aiding in differential diagnosis and monitoring disease progression. In summary, imaging has been instrumental in identifying structural and functional abnormalities, however, the contribution of imaging to understanding MSA’s pathophysiology is limited to confirmation of the marked presence of neuroinflammation. Alpha-synuclein PET tracers, which are currently under development, may provide additional in vivo insights into the patterns of accumulation and propagation of alpha-synuclein in patients with MSA.

Future research efforts should focus on improving the early diagnosis of MSA, particularly in its prodromal stages. This includes the development and validation of neuroimaging markers capable of detecting MSA before the onset of clinical symptoms. Alpha-synuclein PET tracers show promise in this area and warrant further investigation (Smith et al. 2023; Endo et al. 2024; Dong et al. 2025; Goto et al. 2025). Conducting large-scale, longitudinal studies using advanced neuroimaging techniques will be crucial for understanding the progression of MSA. These studies should aim to identify reliable biomarkers that can be utilized in clinical trials for disease-modifying interventions and combining different neuroimaging modalities, such as MRI, PET, and SPECT, may provide a more comprehensive understanding of MSA pathology. Further development and application of machine learning algorithms for automated image-based analysis can enhance diagnostic precision and enable observer-independent assessment of neurodegeneration patterns in MSA. By focusing on these future directions, researchers can advance the understanding of MSA, improve early diagnosis, and develop effective treatments to slow or halt disease progression.
